# The Wafer-Level Integration of Single-Crystal LiNbO_3_ on Silicon via Polyimide Material

**DOI:** 10.3390/mi12010070

**Published:** 2021-01-09

**Authors:** Xiangyu Yang, Wenping Geng, Kaixi Bi, Linyu Mei, Yaqing Li, Jian He, Jiliang Mu, Xiaojuan Hou, Xiujian Chou

**Affiliations:** 1 Science and Technology on Electronic Test and Measurement Laboratory, North University of China, Taiyuan 030051, China; s1806068@st.nuc.edu.cn (X.Y.); bikaixi@nuc.edu.cn (K.B.); s1806120@st.nuc.edu.cn (Y.L.); drhejian@nuc.edu.cn (J.H.); mujiliang@nuc.edu.cn (J.M.); houxiaojuan@nuc.edu.cn (X.H.); chouxiujian@nuc.edu.cn (X.C.); 2 School of Mechanical Engineering, North University of China, Taiyuan 030051, China; mly81@163.com

**Keywords:** LiNbO_3_ single crystalline, low-temperature bonding, oxygen plasma activation, polyimide material, radiation-hardened properties, low-temperature tolerance

## Abstract

In situ measurements of sensing signals in space platforms requires that the micro-electro-mechanical system (MEMS) sensors be located directly at the point to be measured and in contact with the subject to be measured. Traditional radiation-tolerant silicon-based MEMS sensors cannot acquire spatial signals directly. Compared to silicon-based structures, LiNbO_3_ single crystalline has wide application prospects in the aerospace field owing to its excellent corrosion resistance, low-temperature resistance and radiation resistance. In our work, 4-inch LiNbO_3_ and LiNbO_3_/Cr/Au wafers are fabricated to silicon substrate by means of a polyimide bonding method, respectively. The low-temperature bonding process (≤100 °C) is also useful for heterostructure to avoid wafer fragmentation results from a coefficient of thermal expansion (CTE) mismatch. The hydrophilic polyimide surfaces result from the increasing of -OH groups were acquired based on contact angle and X-ray photoelectron spectroscopy characterizations. A tight and defect-free bonding interface was confirmed by scanning electron microscopy. More importantly, benefiting from low-temperature tolerance and radiation-hardened properties of polyimide material, the bonding strength of the heterostructure based on oxygen plasma activation achieved 6.582 MPa and 3.339 MPa corresponding to room temperature and ultra-low temperature (≈ −263.15 °C), which meets the bonding strength requirements of aerospace applications.

## 1. Introduction

Silicon-based micro-electro-mechanical system (MEMS) sensors have been widely used in the aviation and aerospace research field due to their high accuracy, low power consumption and tight integration [1,2,3,4]. In all of these space applications, the functional materials used in MEMS sensors need to withstand high levels of radiation including short wavelength, energetic electromagnetic radiation, and notably gamma rays and X-rays, together with high energy particles and so on [5,6,7,8,9]. The harsh environment poses a threat to traditional silicon-based functional materials, and it is impossible to acquire sensing signals by in situ measurements on a space platform without a radiation hardening process [10,11]. Researchers urgently need to find some new functional materials with intrinsic resistance to irradiation for aerospace applications. As a synthetic crystal material with excellent corrosion resistance, low-temperature resistance and radiation resistance, LiNbO_3_ single crystals exhibit a bright prospect for space platform applications [12,13,14]. Generally, traditional methods including magnetron sputtering and pulsed laser deposition can only obtain polycrystalline or poor-quality LiNbO_3_ films [15,16,17]. However, it is hard to acquire low lattice defects and high precise stoichiometry ratios by the above methods, which lead to the significant deterioration of thin film properties. Therefore, it is vital to realize bonding of single crystal LiNbO_3_ and silicon wafers. 

In recent years, some direct or indirect bonding methods have been developed to transfer single crystalline LiNbO_3_ film onto the silicon-based substrate for solving the problem of compatibility between high-quality piezoelectric materials and silicon-based substrates [18,19,20,21,22,23]. In the high temperature bonding process, there is a fragmentation phenomenon between LiNbO_3_ and Si wafers because of their thermal coefficient mismatch (CET ratio of 6:1 for LiNbO_3_/Si) [20]. The low-temperature bonding of single-crystal LiNbO_3_ wafers on silicon substrates is turning out to be a research hotspot. Wu et al. reported a hydrophilic direct bonding, and the method was used on the fabrication of LiNbO_3_/silicon heterostructure [21]. Nevertheless, the method puts forward a higher request to the cleanliness and smoothness of the bonding surface. Its low bonding strength (≈ 2.5 Mpa) is also difficult to ensure the accuracy and stability of the MEMS sensors in the harsh environment of space. Takagi H et al. developed a method to bond LiNbO_3_ wafer to Si substrate by argon-beam surface activation bonding (SAB) [22]. Although a high-strength bonding of the LiNbO_3_/silicon heterostructure was acquired based on an SAB method, the heterostructure must be worked under ultrahigh vacuum conditions to prevent the activated surface from reoxidation. The lab equipment mentioned in the paper is only produced by Japan, and the high price of equipment has limited its wider application. Different from the above methods, adhesive indirect bonding technology has the ability to finish high strength bonding without strict requirements on the bonding surface. Xiaoyuan et al. presented an indirect bonding method of LiNbO_3_/Si heterostructure bonding via benzocyclobutene (BCB) adhesives [23]. The bonding wafers exhibit a high bonding strength of 8.78 MPa. But the bonding wafers have an obvious warpage of about 15 mm, making the bonded pairs unstable. In most cases, the LiNbO_3_/BCB/Si bonding method in the literature is focused on a small-sized sample (1 cm × 1 cm), and wafer-level bonding is rarely reported. Besides this, there is also no relevant report on the tolerance of total dose irradiation of BCB, which is not enough to address and satisfy the strict requirements in the aerospace market. Despite the above encouraging developments, low-temperature and high strength wafer-level bonding methods are urgently needed to be explored to fully convince the space community of its potential benefits. 

PI materials (the abbreviation of polyimide ) have some advantages in wafer-level bonding for space applications, such as excellent dielectric properties (with a dielectric constant of about 3.4 and a dielectric loss of 10^−3^), good endurance at low temperatures (≥ 4 K), and strong antiradiation damaging features [24,25,26]. Moreover, the PI material with inherently rad-hard are proposed to accomplish low-temperature integration of single-crystal LiNbO_3_ wafers on silicon substrates in our work. Water vapor and solvent volatilizes are produced during the curing process of PI, which leads to holes in the bonding interface [27]. By controlling temperature and time of PI soft baking, an intermediate layer with a certain degree of curing is obtained, which is useful for final bonding. In order to ensure that PI film can be completely attached to the wafer surface, PI solution was spin-coated on the LiNbO_3_ wafer and silicon substrate. Then the PI film was activated by plasma to achieve pre-bonding. The pre-bonded wafer pairs were stored at room temperature in air for 24 h and were then annealed at 100 °C for 15 hours to complete the bonding. A z-cut single crystal LiNbO_3_ with an Au electrode was also used for wafer bonding, and Si-PI-Pt-LN 4-inch samples with no holes in the bonding interface were obtained. The bonding strength of the heterostructure based on oxygen plasma activation achieved 6.6 MPa and 3.339 MPa corresponding to room temperature and ultra-low temperature (≈ −263.15 °C), respectively. A model combining the oxygen plasma activated pre-bonding and the PI bonding procedures was also provided to explain the bonding mechanism by the characteristics of the surface and bonding interface.

## 2. Materials and Methods

### 2.1. Sample Preparation

We used 4-inch Z-cut LiNbO_3_ wafer produced by commercial pulling methods and 4-inch n-type doped [100] Si wafer for wafer bonding. The wafer thickness was about 500 μm. The Cr/Au/Cr layer was deposited as an electrode layer on the flat lithium niobite wafer using a magnetron sputtering technique. During the magnetron sputtering deposition, the cleaned lithium niobite substrate was put in the magnetron sputtering chamber (EXPLORED, America) and the Cr/Au/Cr (20 nm/150 nm/20 nm) layer was deposited at a 500 W power. Prior to spin-coating the PI, the surface cleaning of silicon was carried out according to the RCA cleaning method. The lithium niobite wafer was cleaned only using an acetone solution and an anhydrous ethanol solution due to the instability of the Cr/Au/Cr layer grown by magnetron sputtering. After the above cleaning processes, all the samples were rinsed using de-ionized water and blow-dried using high pure nitrogen. 

After cleaning, PI (commercial name PI-5100 that sold by Feynman Technology Co, Ltd., Raipur, India) bonding glue was spin coated on both lithium niobite samples and the silicon substrate with 3500 rpm for 40 s. The thickness of the PI film was about 3.11 μm. The samples were divided into four groups to obtain the best pre-baking temperature. The first group (A) was baked at 80 °C for 1 min and the second group (B) was baked at 80 °C for 30 min before bonding experiments. In order to fully volatilize the solvent and achieve a certain degree of solidification of PI (PI will produce a by-product H_2_O molecules during curing), we used gradient baking in the third and fourth sets of experiments for soft baking. The third group (C) was baked at 80 °C for 30 min and at 150 °C for 30 min. The fourth group (D) was baked at 80 °C for 30 min, at 150 °C for 30 min, and at 250 °C for 30 min.

**Figure 1:**
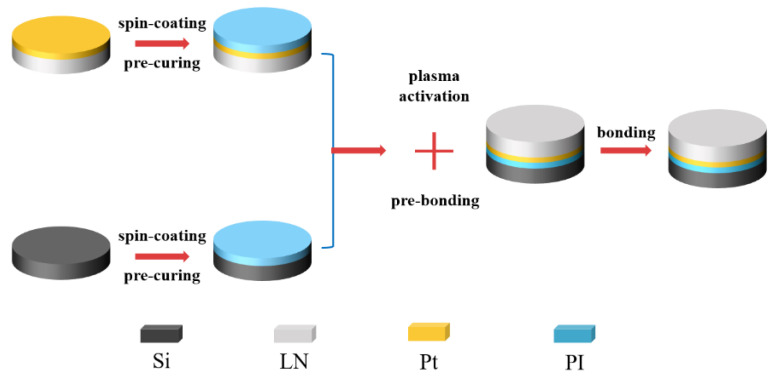
Fabrication process of Lithium Niobate single-crystalline thin film.

As a next step, all the samples were introduced in the PVA-Tepla-loN40 plasma system and activated by a oxygen plasma sputtering samples surface. Plasma activation was conducted when the chamber was evacuated to 150 mTorr. The plasma was generated by a discharge power of 250 W at the gas flow rate of 500 sccm. The activation time was 90 s. After that, the two wafers were taken out of the plasma chamber and brought into contact at room temperature in air. After alignment, by applying a slight pressure at the center, the bonded area spreads laterally over a large area within a few seconds. In order to improve the bonding strength and remove the unbonded area, the pre-bonded wafer pairs were put into a wafer bonder (EVG510, Austria) with an ultimate vacuum of 1 × 10^−5^ Pa and applied a force of 4000 N for 30 min. The pre-bonded wafer pairs were stored at room temperature in air for 24 h to saturate the pre-bonding strength. Then the pre-bonded pairs were annealed at 100 °C for 15 hours to complete the bonding. The preparation flow chart of lithium niobate single crystal film is shown in Figure 1.

### 2.2. Characterization and Measurements

A contact angle testing instrument with a side-camera imaging system (DSA100, KRUSS, Hamburg, Germany) was used to obtain the water contact angle. The surface chemical states of the PI film after activation were characterized by X-ray photoelectron spectroscopy (XPS: ESCALAB 250Xi, Thermo Fisher, Waltham, Massachusetts, America). The tensile tester (Extensograph AI-3000S, GOTECH, Taichung, Taiwan, China) was applied to test the bonding strength. In this test, a 1.023 mm-wide bonded sample was pulled at the rate of 200 mm/min, and the average force was calculated to yield the bonding strength. The bonded interfaces of the bonded samples were identified by scanning electron microscopy (SEM: SUPRA-55, ZEISS, Oberkochen, Badensko-Wuertembersko, Germany).

## 3. Results and Discussion

**Figure 2:**
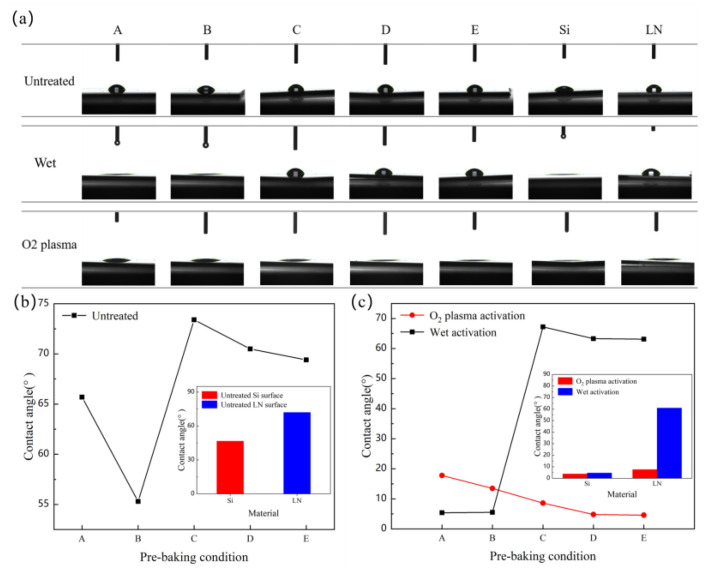
(**a**) The shape of the water drop on the surface in each of the test points. (A. Pre-baking at 80 °C for 1 min; B. Pre-baking at 80 °C for 30 min; C. Pre-baking at 80 °C for 30 min and 150 °C for 30 min; D. Pre-baking at 80 °C for 30 min and 150 °C for 30 min and 250 °C for 30 min. E. Fully cured.) (**b**) Contact angle of PI film versus pre-baking condition. The inset shows the contact angle of LN and Si. (**c**) Contact angle of PI film versus pre-baking condition after wet chemical activation and O_2_ plasma activation. The inset shows the contact angle of LN and Si.

Wafer surface energy is one of the important parameters that determines the success of wafer bonding. According to the wafer bonding conditions of H.H. YU and Z. SUO (Equation (1)), the greater wafer surface energy will cause the bonding between the two wafers to become easier to achieve spontaneously [28]. The surface energy can be directly reflected by the surface contact angle, which in the smaller contact angle means a greater surface energy [29]. When the contact angle is less than 90°, the surface of the wafer is hydrophilic, which is easily combined with water molecules in the air through hydrogen bonds. In order to study the effect of different pre-baking conditions on the wettability of the wafer surface, we carried out water contact angle examination on the PI films. As shown in Figure 2b, the contact angles of the PI films obtained under different pre-baking conditions are all less than 90°. When the pre-baking temperature is lower than the minimum curing temperature (150 °C), PI films exhibit higher hydrophilicity, which indicates that the polar solvent has not completely evaporated. When the PI film was baked at 80 °C for 30 min and 150 °C for 30 min, the contact angle rose from 53.7° to 73.4°, which indicated that the polar solvent had evaporated completely. The contact angle gradually decreases with the baking temperature increasing. At the same time, we tested the contact angle of the LN and Si wafer surfaces, and the results showed that the Si surface (46.6°) is more hydrophilic than the LN surface (72°).

(1) U-Γ·A<0

where *U* is the elastic strain energy accumulated after the wafers are bonded, *Γ* is the energy required per unit bonding area during bonding and *A* is the bonding area.

Figure 2c shows the contact angle of the film after wet activation and O_2_ plasma activation to understand the affinity of film surfaces by wet and dry treatments. O_2_ plasma treatment has significant effects on the surface of the film, which is manifested as a significant decrease in the contact angle of the film surface, which is caused by the increase in the density of surface polar groups [30]. When the pre-baking condition is D (at 80 °C for 30 min, 150 °C for 30 min and 250 °C for 30 min), the film surface contact (4.8°) angle is lower than 5°, which means greater surface energy and meets the surface requirement of bonding. Moreover, the film at this time has achieved a certain degree of curing, but the cross-linking between molecules is not obvious. This result lays a good foundation for the success of subsequent bonding. The contact angle of LN wafer surface by plasma activation is 7.6°. During the wet process, we found that when the pre-baking temperature is lower than 150 °C, the film dissolves in the solution, indicating that the main component of the film at this time is polyamide acid, and the contact angle at this time is the contact angle of the wafer surface. Compared with plasma treatment, the effect of wet activation is not very significant. Since the effect of activation will decrease over time, the sample needs to be pre-bonded immediately after plasma activation [31].

To fully understand the bonding mechanism, chemical states of PI film surfaces treated in different ways were analyzed by XPS. Table 1 shows the elemental percent on the PI film surfaces untreated or treated in different ways, which were calculated based on the peak intensity of each element. The atomic percentage of oxygen on the PI film surfaces treated with O_2_ plasma was significantly increased compared with untreated PI film surfaces. This is due to the introduction of oxygen-containing functional groups on the surface of the PI film. Meanwhile, in the process of O_2_ plasma treatment of PI films, the activated species destroys the C-C chemical bond on the surface of PI films, which reduces the percentage of carbon. The percentage of elements on PI film surfaces after wet processing did not change significantly, which also verifies the resistance of PI at this time to strong alkali corrosion.

**Figure 3:**
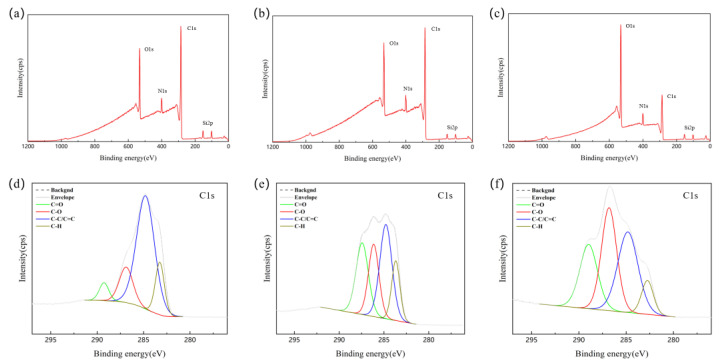
XPS survey spectra and high-resolution spectra on the peak of C1s of PI film surfaces after pre-baking at 80 °C, 30 min; 150 °C, 30 min; 250 °C, 30 min. (**a**,**d**) untreated surface, (**b**,**e**) wet treated, (**c**,**f**) treated with O_2_ plasma.

Figure 3a shows the XPS survey spectra of untreated PI film pre-baked at 80 °C for 30 min, 150 °C for 30 min, and 250 °C for 30 min. According to the survey spectra, there are mainly C1s, N1s, and O1s peaks on PI film surfaces. In addition, Si 2p peaks were observed, which were caused by silicon impurities remaining in the chamber. According to Figure 3b, it can be seen that the spectrum of the PI film after the wet treatment has not changed significantly. The film does not react with strong bases. As shown in Figure 3d–f, high-resolution XPS fine scans were performed on C1s. It can be seen that the C1s spectra can be fitted as four subpeaks, corresponding to C-H (about 283.3 eV), C-C/C=C (about 284.8 eV), C-O (about 286.8 eV), C=O (about 289.2 eV). We have observed that the strength of the C-O and C=O chemical bonds increased significantly after O_2_ plasma treatment, and increased to a certain extent after wet treatment. Due to the increase in the amounts of polar groups such as -OH, CO, and COO- on the surfaces, the treated PI film surface shows unique high polarity and low dispersion, which greatly improves the surface adhesion. XPS analysis confirmed the improvement of the wettability of the film surfaces treated with O_2_ plasma.

**Figure 4:**
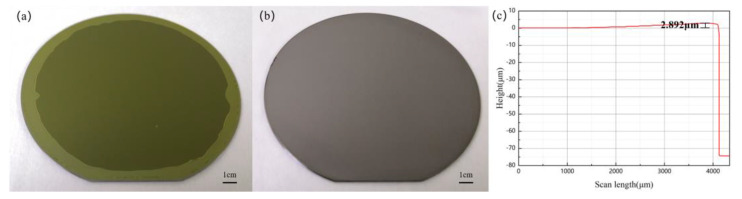
(**a**) Optical photograph of LN/PI/Si bonded pairs. (**b**) Optical photograph of LN/Au/PI/Si bonded pairs. The inset shows the optical photograph of 1 cm × 1 cm bonded pairs after annealing at 250 °C for 2 h. (**c**) Step height of LN/Au/PI/Si bonded pairs surface.

Although PI has an excellent performance, there is a by-product (H_2_O) during the curing process. The by-product will cause holes in the bonding interface. Therefore, we need to soft bake the PI to make the solvent fully volatilize and reach a certain degree of curing. In this way, few by-products were generated during the curing process of PI, so that no holes appeared in the bonding interface. Based on the above discussion, the PI film baked at 80 °C for 30 min, 150 °C for 30 min, and 250 °C for 30 min meets this condition. And after O_2_ plasma activation, it shows high hydrophilicity. The bonding process was carried out after PI was prebaked at 80 °C for 30 min, 150 °C for 30 min, and 250 °C for 30 min. Figure 4a presents the bonded pairs of LN wafer without a metal layer and Si wafer after indirect bonding. Due to the transparency of lithium niobate, the resulting bonding interface can be seen by looking through the lithium niobate layer. Evaluating the integrity of the bonding was realized easily by a visual inspection of voids formed between the LN wafer and the Si wafer. Seen from Figure 4a, there are almost no bubbles and voids at the bonded interface, and no cracks can be observed at the interface, but there is an unbonded area at the edge. It was mainly caused by the protruding edges of the PI film and the polycondensation of the PI bonding glue. After the PI bonding glue was spin-coated on the surface of the wafer, the bonding glue will accumulate on the edge of the wafer, which causes the glue layer on the edge to be higher than the glue layer on the inner side and is uneven. Due to the high residual solvent content of the edge of the bonding glue, the solvent volatilization is insufficient and the cyclization reaction proceeds slowly, which will adversely affect the subsequent activation and bonding process. The illustration shows that the bonding interface after PI is fully cured and is still complete and uniform. Figure 4b presents the bonded sample of the LN wafer with a metal layer and an Si wafer after indirect bonding. The indirect wafer bonding was carried out for the PI (about 3.11 μm)/Au (about 192 nm)/lithium niobate flat wafer and PI (about 3.11 μm)/silicon flat wafer to fabricate the lithium niobate/polyimide/Au/silicon structure. The electrode layer was still complete and uniform after the wafer bonding by visual inspection. The post-bond warpage is about 2.89 μm, which is extremely low and can guarantee the subsequent application of the bonded pairs, as shown in Figure 4c.

**Figure 5:**
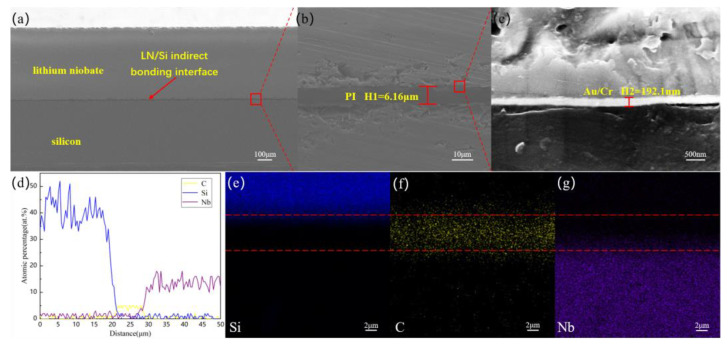
(**a**–**c**) SEM image of the LN/Au/PI/Si bonding interface. (**d**) EDX-Line spectra of the bonding interface. (**e**–**g**) EDX mappings of the bonding interface.

Figure 5a–c illustrates the SEM cross-sectional views of the LN/Au/PI/Si indirect bonded sample. Each layer of the indirect bonding interface can be clearly observed and thickness is uniform, which is the LN layer, the Cr/Au/Cr electrode, the PI layer, and the Si substrate severally from top to bottom. The thickness of the PI film was measured by SEM, and its thickness was about 6.16 μm. Therefore, we speculate that the thickness of the PI film spin-coated by Spin Coater for 3500r/min was approximately 3.08 μm. The thickness of the electrode layer with the structure of Cr/Au/Cr is about 192 nm. The 150 nm Au layer is used as the electrode layer, and the 20 nm Cr layers are used as the adhesion layer. It can be seen that the bonding interfaces are tightly and clearly visible. The EDX-Line scanning spectra depicts the distributions of Si, C, and Nb atoms across the bonding interface, as illustrated in Figure 5d. Because the sample needs to be sprayed with gold before the test, the reference value of the Au peak is not significant, so the Au element peak is not displayed. The width of the C peak is about 7 μm, which is slightly larger than the measured value of the SEM image, which confirms the diffusion of the C element. In addition, the element distribution of the bonding interface was identified by EDX mapping, as presented in Figure 5e–g. There was an interdiffusion that occurred between C, and Si, Nb elements and the atoms diffuse and migrate to form atomic-level connections at the interface.

**Figure 6:**
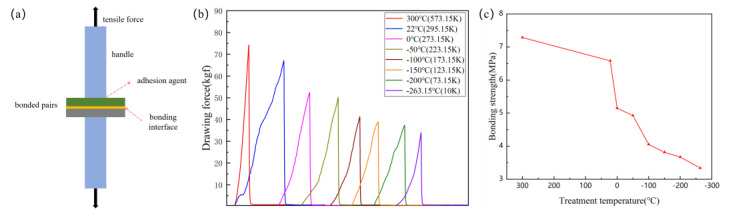
(**a**) Schematic diagram of bonding strength test structure; (**b**) Stretching resistance; (**c**) bonding strength of LiNbO_3_/Si bonded pairs after different temperature treatments for 24 h.

Bonded pairs were divided into 1 cm × 1 cm for the tensile test. The samples were kept at low temperature for 24 h to verify the low temperature resistance of the LN/PI/Si structure. This test has not been reported before. As shown in Figure 6a, the bonded samples (1 cm × 1 cm) were fixed on the handles with epoxy glues and loaded into the vertical fixtures. The bonding strength of the untreated sample is 6.582 Mpa. The bonding strength of the sample treated at 300 °C is slightly improved, which is 7.287 Mpa. However, the bonding strength of the sample after the low temperature treatment is reduced, as shown in Figure 6c. In a low temperature environment, the mechanical properties of PI decline, and its adhesion to LN or Si decreases. In the process of low temperature treatment, the residual stress at the bonding interface gradually accumulates. The bonding strength of the sample treated at −263.15 °C (10 K) for 24 h is 3.339 Mpa, which still meets the minimum requirements of the device for bonding strength. At the same time, we also tested the bonding strength (11.379 Mpa) of the bonded sample after the PI was fully cured.

**Figure 7:**
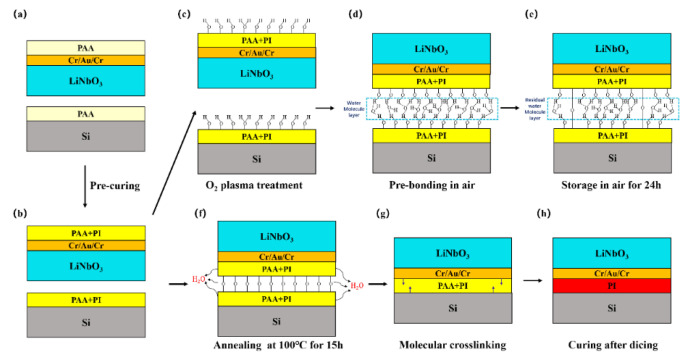
The schematic drawings for LiNbO_3_/Au/PI/Si bonding mechanisms. (**a**) Pre-curing; (**b**) Pre-baking; (**c**) O2 plasma treatment; (**d**) Pre-bonding in air; (**e**) Storage in air for 24 h; (**f**) Annealing at 100 °C for 15 h; (**g**) molecular crosslinking; (**h**) Curing after dicing.

Figure 7 clarifies the bonding mechanism of polyimide/Au/lithium niobate wafer and polyimide/silicon wafer bonding. PI reaches a certain degree of curing after pre-baking (Figure 7b). Combining the above discussion and analysis of the bonding surface and the bonding interface, as well as the previously published reports [32,33,34,35], we can get the effect of O_2_ plasma on the surface of the PI film: (1) removing surface contaminants; (2) increasing the number of -OH (hydroxyl) groups pre unit area on the surface; (3) changing the surface structure and improving the diffusion capacity of water and gas on the surface. The experimental results show that the PI film becomes smoother and exhibits more significant hydrophilicity after O_2_ plasma treatment (Figure 7c). The two wafers are in contact with each other in the air at room temperature to complete the pre-bonding (Figure 7d). At this time, the two wafers are primarily held together by van der Waals forces and hydrogen bonds between monolayers of water molecules and the -OH groups. In order to improve the bonding strength, we sent the pre-bonded sample into the wafer bonder and kept it under 4000 N force for 30 min, which provided a closer contact between the two surfaces. Then bonded wafer pairs are stored at room temperature in air for 24 h. During this process, a part of the water molecules in the bonding interface evaporates [36], which pulls the C-OH groups in the bonding interface very close together, thereby increasing the bonding strength (Figure 7e).

(2) $$C\text{-}\mathrm{OH}\;+\;C\text{-}\mathrm{OH}\;\to {\;C\text{-}O\text{-}C\;+\;H}_{2}O$$

After that, the bonded sample are annealed at 100 °C for 15 h to obtain a stronger bonding strength. During the annealing process, a dehydration reaction occurs at the bonding interface (Figure 7f). The molecules at the bonding interface diffuse each other, and the molecules are cross-linked with each other, and the hydrogen bonds are transformed into C-O-C covalent bonds. During the annealing process, water molecules tend to diffuse along the interface. When there are no water molecules at the interface where the two wafers are joined, a stable C-O-C covalent bond will be formed. The formation of C-O-C covalent bonds will promote intermolecular crosslinking (Figure 7g). Therefore, the bonded pairs can obtain great bonding strength after bonding, which fully meets the requirements of chemical mechanical thinning and polishing for bonding strength. The bonded wafer pairs are annealed after dicing to achieve complete curing of the PI. 

**Figure 8:**
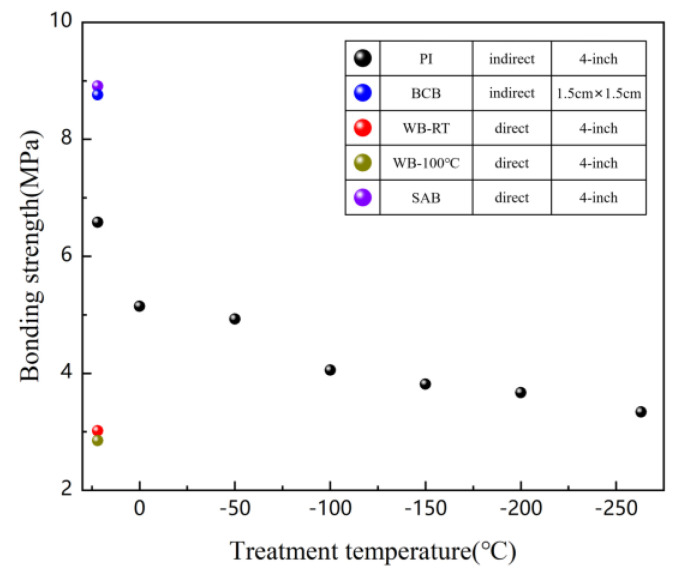
Bonding strength of LiNbO_3_ single crystal and silicon substrate by the PI bonding method, the BCB bonding method, the conventional wafer bonding(denoted WB), and the room-temperature SAB.

The bonding strength of samples prepared by different methods were compared, as shown in Figure 8. Although the bonding pairs prepared by BCB-based bonding technology exhibit a high bonding strength, it is currently only used for small area bonding. Wafer-level LN/BCB/Si bonding pairs have a very high warpage of about 15 mm, making the pairs unusable. There is no report on the anti-radiation and low temperature resistance of BCB. The strength of the LN/Si bonding pairs prepared by the conventional wafer bonding method was small. High-strength LN/Si bonding pairs can be prepared by SAB (surface activated bonding) at room temperature, but the equipment to implement this method is very expensive. The device is currently only reported in Japan. The PI-based bonding method proposed in this paper can also achieve wafer-level bonding with high strength. It still maintains high bonding strength at low temperatures.

## 4. Conclusions

In this paper, the wafer-level integration of single-crystal LiNbO_3_ on silicon is achieved based on oxygen plasma activation and polymer bonding technology under a low temperature. The high bonding strength (6.582 Mpa) can withstand the stress caused by the harsh chemical-mechanical thinning and polishing process. The tensile test results of the samples treated at a low temperature shows that the samples still maintain a strong bonding. For example, the bonding strength of the sample treated at −263.15 °C for 24 h is 3.339 Mpa. SEM results show that the bonding interface is defect-free and uniform, and a clear metal layer can be seen. The surface properties and chemical composition of the plasma-treated PI film were studied by contact angle and XPS spectroscopy. The results show that oxygen plasma can be completely used for the chemical modification of the PI film surface, which fully meets the requirements of pre-bonding. Based on the surface and bonding interface characteristics, a model combining the plasma-activated pre-bonding and the polymer-bonding procedures is proposed to explain the bonding mechanism. The technology is of great significance to promote the application of high-performance LiNbO_3_-on-silicon devices in the harsh environment of space with low temperatures and strong radiation.

## Figures and Tables

**Table 1:** The atomic percentage on PI surface with/without treatment.

Sample	Atomic Percent (at.%)			
	C	N	O	Si
None treatment	75.03	5.63	15.47	3.88
Wet treatment	75.37	6.18	16.03	2.41
O_2_ plasma treatment	51.71	7.65	36.72	3.93

## Data Availability

Data sharing is not applicable to this article.
